# Editorial: Spatial and Temporal Perception in Sensory Deprivation

**DOI:** 10.3389/fnins.2021.671836

**Published:** 2021-03-30

**Authors:** Irene Senna, Luigi F. Cuturi, Monica Gori, Marc O. Ernst, Giulia Cappagli

**Affiliations:** ^1^Department of Applied Cognitive Psychology, Ulm University, Ulm, Germany; ^2^Italian Institute of Technology (IIT), Genoa, Italy; ^3^Neurological Institute Foundation Casimiro Mondino (Istituto di Ricovero e Cura a Carattere Scientifico), Pavia, Italy

**Keywords:** sensory deprivation, cortical plasticity, spatial perception, temporal perception, visual impairment, hearing impairment, motor impairment, rehabilitation

The Research Topic aimed at providing new insights into the impact of sensory deprivation on spatio-temporal abilities and their subtending cortical circuits. The Research Topic attracted a wide range of submissions across the spectrum of this theme, and overall, all the submitted papers fall within one of the following topic contributions: (a) papers identifying impaired/preserved abilities after a sensory loss/deprivation; (b) papers investigating cortical plasticity and reorganization mechanisms following sensory loss/deprivation; (c) papers presenting newly developed tools to assess and/or train spatial impairments resulting from sensory loss/deprivation. With this editorial, we intend to discuss the findings of the submitted contributions within the broader context of the literature on the theme by considering the three above-mentioned main contribution categories.

## Impaired vs. Preserved Perceptual Functions After Sensory Loss/Deprivation

Overall, five out of the six papers in this category demonstrated that sensory loss/deprivation leads to perceptual and sensorimotor impairments rather than preserved abilities. Wu et al. demonstrated that long-term abnormal binocular visual experience causing intermittent but recurrent eye misalignment (intermittent exotropia) alters distance stereoscopic acuity (Hatt et al., 2007; Zhou et al., 2019), thus impairs three-dimensional depth perception. The Authors demonstrated that patients with intermittent exotropia require longer times for optimal stereoacuity, arguing that more extended temporal integration might be caused by a longer time needed for binocular cells to integrate the signals from two eyes. This new finding sheds light on the importance of including the temporal dimension of stimulus presentation in stereopsis assessment and rehabilitation training. Luo et al. demonstrated that a clinical condition characterized by progressive visual acuity decrease and progressive peripheral visual field loss (retinitis pigmentosa) affects general visual information processing and specific visuo-spatial and visuo-attentional capabilities.

Similarly, Martolini et al. demonstrated that children with impoverished visual experience from birth (low vision) acquire the ability to represent space based on external frames of reference (“allocentric”) rather than on body-centered cues (“egocentric”) much later compared to sighted peers. Such finding is in line with previous evidence showing that vision is necessary to guide the development of spatial abilities (Thinus-Blanc and Gaunet, 1997; Eimer, 2004; Iachini and Ruggiero, 2010; Pasqualotto and Proulx, 2012; Cappagli and Gori, 2016; Voss, 2016; Cappagli et al., 2017) and that long-term early-onset visual impairment might compromise such development. Scotto et al. reported that short-term sensorimotor deprivation causes impairments in motor control by disrupting the spatiotemporal structure of the pointing movements performed after the deprivation. More specifically, they showed that when healthy individuals immobilize their right limb for 24 h, not only their overall motor performance decreases as previously shown (Huber et al., 2006; Moisello et al., 2008; Bassolino et al., 2012; Bolzoni et al., 2012), but also both early and late kinematic parameters (corresponding to feedforward and feedback processes of motor control, respectively) are altered. This evidence indicates that short-term sensorimotor deprivation alters motor control both at an early step (feedforward control), impairing the ability to predict future actions' sensory consequences, and at a later step (feedback control), and the ability to correct reaching movements toward the target. Since visual cues strongly influence feedback control of movements (Sarlegna et al., 2004, 2007; Saunders and Knill, 2004; Sarlegna and Sainburg, 2009), future research should investigate whether visual feedback during movement can overcome the motor impairments observed after prolonged limb immobilization.

Sharp et al. demonstrated that congenital deafness impairs the development and maintenance of overt oculomotor behavior, suggesting that a hearing impairment can affect the non-deprived visuo-motor domain. Contrary to the other studies presented above, which investigated intra-modal consequences of sensory loss/deprivation, this study directly assessed the link between auditory experience and the development of visual functions. Such evidence corroborates recent hypotheses suggesting the existence of cross-sensory integration and calibration mechanisms (Gori et al., 2010; Morrone, 2010; Gori, 2015; Dekker and Lisi, 2020), thanks to which the most accurate sensory modality for a specific task (e.g., hearing for temporal discrimination) dominates and guides the development of the others. According to this view, it might be hypothesized that hearing would have a role in the control of eye movements. This finding fits well within the literature demonstrating altered eye movement control in the deaf (Bottari et al., 2012). Further studies should investigate how auditory loss impacts crossmodal reorganization in terms of functional change (Cardin et al., 2020). The only study that revealed preserved abilities after sensory deprivation is the one by Chen et al., showing that short-term visual deprivation in one eye does not impair the ability to judge the temporal synchrony of visual stimuli presented after the deprivation in dichoptic and monocular conditions. Contrarily to previous behavioral and electrophysiological/neuroimaging studies showing that monocular deprivation causes a shift in perceptual ocular dominance (Lunghi et al., 2011; Zhou et al., 2013, 2014; Kim et al., 2017; Başgöze et al., 2018; Min et al., 2018) and increased response of the deprived eye vs. a decreased response of the non-deprived eye (Lunghi et al., 2015a,b; Zhou et al., 2015; Chadnova et al., 2017; Binda et al., 2018), this study indicates that such kind of visual deprivation does not influence the temporal processing of visual information. Factors such as the type of task (e.g., binocular rivalry vs. phase combination), the assessed perceptual domain (e.g., spatial vs. temporal processing), and the duration of visual deprivation might underlie such discrepancy.

## Cortical Plasticity After Sensory Loss/Deprivation

Sensory loss or deprivation typically induces significant reorganization in sensory cortices (Rauschecker, 1995; Bavelier and Neville, 2002; Merabet and Pascual-Leone, 2010; Ricciardi and Pietrini, 2011). It has been argued that crossmodal plasticity may take the form of functional preservation, where cortical regions preserve their function but adapt to process sensory input in a different modality. Or it can result in functional change, where cortical regions change also their function, typically switching from sensory processing to higher order cognition (Cardin et al., 2020). Such plastic reorganization often subtends compensatory mechanisms, which can enable even normal or close-to-normal perceptual abilities. Scurry et al. investigated possible differences between early deaf and typical hearing individuals in a visual-tactile temporal judgment task. Differences in performance were expected, since audition is believed to provide a necessary framework for developing sensitivity to temporal information (Burr et al., 2009; Conway et al., 2009). Surprisingly, the two groups did not differ in their temporal order perceptual performance. However, deaf participants showed enhanced EEG signal strength in both visual and tactile components compared to sighted controls, which indicates compensatory recruitment of auditory and visual areas for visuo-tactile temporal processing. Scurry et al. reported that multisensory areas, such as the right posterior superior temporal sulcus (pSTS), undergo compensatory plasticity. In particular, early deaf individuals showed larger activation of the pSTS compared to healthy controls during tactile motion processing. This activation, which is not accompanied by increased directional tuning, suggests the presence of a more distributed network of neuronal populations involved in tactile motion processing as a consequence of early auditory deprivation. However, in line with the principle of functional preservation, no greater activation of the primary auditory cortex (PAC) was found: audition is predominant in processing temporal features, and visual and tactile temporal tasks lead to PAC activations in the blind (Auer et al., 2007; Bola et al., 2017). This study shows that PAC maintains its temporal processing involvement after a sensory loss without being involved in processing spatial–rather than temporal–tactile aspects. Glick and Sharma demonstrated that early stage mild-moderate age-related hearing loss is associated with cross-modal recruitment of auditory, frontal and prefrontal cortices during visual tasks, suggesting functional changes induced by hearing loss. Significantly, more extensive recruitment of the auditory cortex by vision correlates with more significant hearing loss and lower perceptual and cognitive performance. Moro et al. showed that partial visual deprivation, such as the early loss of one eye, can induce a neuronal reorganization of circuits typically dedicated to binocular vision, resulting in increased brain activation for audio-visual stimuli.

Unfortunately, such cross-modal cortical reorganization can also result in maladaptive outcomes. This process can happen either due to early-onset sensory deprivation or when sensory deprivation or decline occurs later in life. Maladaptive changes led by long-term plasticity are reported by Amadeo et al., who showed that late blind individuals with long time blindness duration present behavioral performance and cortical activations analogous to those shown by early blind individuals. In these participants, temporal cues activate circuits typically responding to spatial cues in both sighted individuals and blind participants with shorter blindness duration. In other words, after many years of blindness, late blind participants start relying on temporal information to build spatial representations, as it happens in early blind individuals (Gori et al., 2013). The fact that many years of late sensory deprivation/decline can lead to maladaptive outcomes highlights the importance of introducing rehabilitation strategies soon after the onset of sensory loss/decline. Notably, the research from Glick and Sharma demonstrates that few months of clinical treatment with hearing aids at an early stage of hearing loss can induce a reversal in the observed cross-modal reorganization of the cortex, accompanied by improved behavioral performance.

## New Tools to Assess and Train Sensorimotor Functions After Sensory Loss/Deprivation

Perceptual impairments following sensory loss/deprivation, such as spatial deficits resulting from visual deprivation, posit the necessity to develop and adapt clinical assessment and training tools to meet the sensory loss population's needs. Specifically, specific tools for visually impaired children are less systematically used and spread than those designed for adults (Gori et al., 2016; Elsman et al., 2019). The need for such solutions has been extensively reported in the literature, but the communication between scientific findings and technological development can still benefit from investigations aiming at developing clinical settings and training strategies. Aprile et al. provided a review of standardized and non-standardized tools in use to assess spatial cognition in visually impaired children by employing other sensory modalities than vision, such as haptic/proprioception and audition. By highlighting the limitation in visual impairment dedicated tools, the Authors mainly focused on the lack of formal and informal assessment methods, and promoted the validation of large-scale application of newly developed tools in the context of pediatric visual impairment.

Tivadar et al. investigated mental rotation abilities in blind participants with a digital haptic technology, which was previously tested with sighted participants. In contrast to sighted participants, visually impaired participants generalized training among letters suggesting the involvement of supramodal processes. In the case of visual loss, such functions can be trained to allow blind participants to make better use of more conceptual than sensory-specific encoding strategies to solve tasks requiring the spatial manipulation of mental representations. Morelli et al. presented a longitudinal study reporting a detailed example of a multisensory rehabilitation intervention leading to improved spatial cognition in a visually impaired child (from 9 months to 11 years of age). The Authors highlighted how early and timely intervention is fundamental to sustain and promote neuropsychomotor development in visual impairment. Rehabilitation is often aided by technological solutions that may improve spatial perception and cognition based on the remaining senses. In this context, sensory substitution devices (SSDs) can effectively enhance spatial competence, such as navigating through space independently. As pointed out in this research topic and in the literature (Cuturi et al., 2016), assessing the blind population is often neglected in technological development. Jicol et al. scrupulously tested potential improvements in spatial navigation tasks with two SSDs: the vOICe (Meijer, 1992), which exploits auditory information and the BrainPort (Bach-y-Rita and Kercel, 2003), which provides participants with tactile information on their tongue about the navigated environment. In one experiment, results from sighted participants showed that the combined use of both SSDs provides no improvement, likely because of task difficulty and sensory overload. In another experiment focusing on integrating auditory and self-motion information in sighted and blind participants, only the latter takes advantage of the vOICe device while navigating on the basis of egocentric and allocentric information.

Chebat et al. provided a comprehensive review on the use of SSDs in the acquisition of spatial competence and brain reorganization in case of blindness. The Authors discuss the brain correlates of spatial navigation strategies and support the notion that a modal processing of space can aid spatial navigation in blind individuals. Regarding future research directions on SSDs, the Authors suggest deepening the study of SSDs employment during the first years of development when brain plasticity is most and great improvement may be expected (Röder et al., 2020; Röder and Kekunnaya, 2021). However, not only SSDs but also everyday technologies might foster spatial cognition in the context of sensory deprivation. Holmer et al. tested whether gaming habit with computer and console games influences visuo-spatial control in deaf individuals. Although gaming experience did not influence hearing individuals performance, deaf individuals benefitted from gaming experience compared to deaf non-gamers, likely by improving visuo-spatial attentional control in the peripheral visual field.

## Author Contributions

IS, LC, and GC wrote the manuscript. IS, LC, ME, MG, and GC revised the manuscript. All authors contributed to the article and approved the submitted version.

## Conflict of Interest

The authors declare that the research was conducted in the absence of any commercial or financial relationships that could be construed as a potential conflict of interest.
